# Targeting the Oncogenic E3 Ligase Skp2 in Prostate and Breast Cancer Cells with a Novel Energy Restriction-Mimetic Agent

**DOI:** 10.1371/journal.pone.0047298

**Published:** 2012-10-12

**Authors:** Shuo Wei, Po-Chen Chu, Hsiao-Ching Chuang, Wen-Chun Hung, Samuel K. Kulp, Ching-Shih Chen

**Affiliations:** 1 Division of Medicinal Chemistry and Pharmacognosy, College of Pharmacy, The Ohio State University, Columbus, Ohio, United States of America; 2 National Institute of Cancer Research, National Health Research Institute, Zhunan, Miaoli County, Taiwan; 3 Institute of Basic Medical Sciences, National Cheng-Kung University, Tainan, Taiwan; University of Kentucky College of Medicine, United States of America

## Abstract

Substantial evidence supports the oncogenic role of the E3 ubiquitin ligase S-phase kinase-associated protein 2 (Skp2) in many types of cancers through its ability to target a broad range of signaling effectors for ubiquitination. Thus, this oncogenic E3 ligase represents an important target for cancer drug discovery. In this study, we report a novel mechanism by which CG-12, a novel energy restriction-mimetic agent (ERMA), down-regulates the expression of Skp2 in prostate cancer cells. Pursuant to our previous finding that upregulation of β-transducin repeat-containing protein (β-TrCP) expression represents a cellular response in cancer cells to ERMAs, including CG-12 and 2-deoxyglucose, we demonstrated that this β-TrCP accumulation resulted from decreased Skp2 expression. Evidence indicates that Skp2 targets β-TrCP for degradation via the cyclin-dependent kinase 2-facilitated recognition of the proline-directed phosphorylation motif ^412^SP. This Skp2 downregulation was attributable to Sirt1-dependent suppression of COP9 signalosome (Csn)5 expression in response to CG-12, leading to increased cullin 1 neddylation in the Skp1-cullin1-F-box protein complex and consequent Skp2 destabilization. Moreover, we determined that Skp2 and β-TrCP are mutually regulated, providing a feedback mechanism that amplifies the suppressive effect of ERMAs on Skp2. Specifically, cellular accumulation of β-TrCP reduced the expression of Sp1, a β-TrCP substrate, which, in turn, reduced Skp2 gene expression. This Skp2-β-TrCP-Sp1 feedback loop represents a novel crosstalk mechanism between these two important F-box proteins in cancer cells with aberrant Skp2 expression under energy restriction, which provides a proof-of-concept that the oncogenic Csn5/Skp2 signaling axis represents a “druggable” target for this novel ERMA.

## Introduction

The ubiquitin-proteasome system plays a pivotal role in regulating key cellular functions through targeted degradation of regulatory proteins, which is initiated by ubiquitination, followed by proteolysis via the 26S proteasome complex. The targeted ubiquitination is mediated through the concerted action of three enzymes: E1 ubiquitin-activating enzyme, E2 ubiquitin-conjugating enzyme, and E3 ubiquitin ligase. Among various E3 ubiquitin ligases, the Skp1-Cul1-F-box protein (SCF) complex represents the largest family, which has received much attention because of its intimate role in regulating cell cycle progression by facilitating targeted degradation of cell cycle-regulatory proteins [1]. The specificity of the SCF ubiquitin ligase for its target proteins is conferred by interchangeable substrate-recognizing subunits, i.e., F-box proteins. More than 70 F-box proteins, each specific for a distinct set of substrates, have been identified in human cells, which provides the basis for the targeted degradation of diverse regulatory proteins during cell cycle progression [2]. Some of these F-box proteins have received much attention in light of their involvement in tumorigenesis, among which S-phase kinase-associated protein (Skp)2 and β-transducin repeat-containing protein (β-TrCP) are especially noteworthy [2]. Substantial evidence indicates that Skp2 acts as an oncoprotein by targeting a wide range of signaling effectors, such as the tumor suppressor p27 [3], for degradation. More recently, it was demonstrated that Skp2 facilitates the activation of Akt through ubiquitination downstream of ErbB receptor signaling in Her2-positive breast cancer [4], and that Skp2, by triggering NSB1 ubiquitination, represents a key component for the Mre11/Rad50/NBS1 complex-mediated activation of ATM in response to DNA double-strand breaks [5]. Consequently, its overexpression has been correlated with poor prognosis in many types of cancers [2]. In contrast, β-TrCP plays an oncogenic or tumor suppressive role in a cellular context-dependent manner considering its diverse substrate spectrum. Although evidence suggests its oncogenic character is mediated through the activation of NF-κB signaling [6], β-TrCP also facilitates the degradation of a wide array of tumor-promoting proteins, including β-catenin, Snail, ATF4, cdc25A, Mcl-1, cyclin D1, and Sp1 [7], [8], [9], [10], [11], [12], [13], thereby suppressing cancer cell proliferation and invasion. Moreover, β-TrCP has been shown to negatively regulate Skp2 expression by facilitating the proteolysis of Emi1 [14], [15]. As Emi1 is an endogenous inhibitor of the anaphase promoting complex/cyclosome E3 ligase (APC/C^Cdh1^), elimination of Emi1 promotes APC/C^Cdh1^-mediated degradation of Skp2 [16], [17]. From a mechanistic perspective, the ability of β-TrCP to facilitate Skp2 degradation through Emi1-APC/C^Cdh1^ signaling underscores the intricate relationship between SCF and APC/C in regulating cell cycle progression [1]. However, in contrast to Skp2, the E3 ligase responsible for β-TrCP degradation remains undefined.

**Figure 1:**
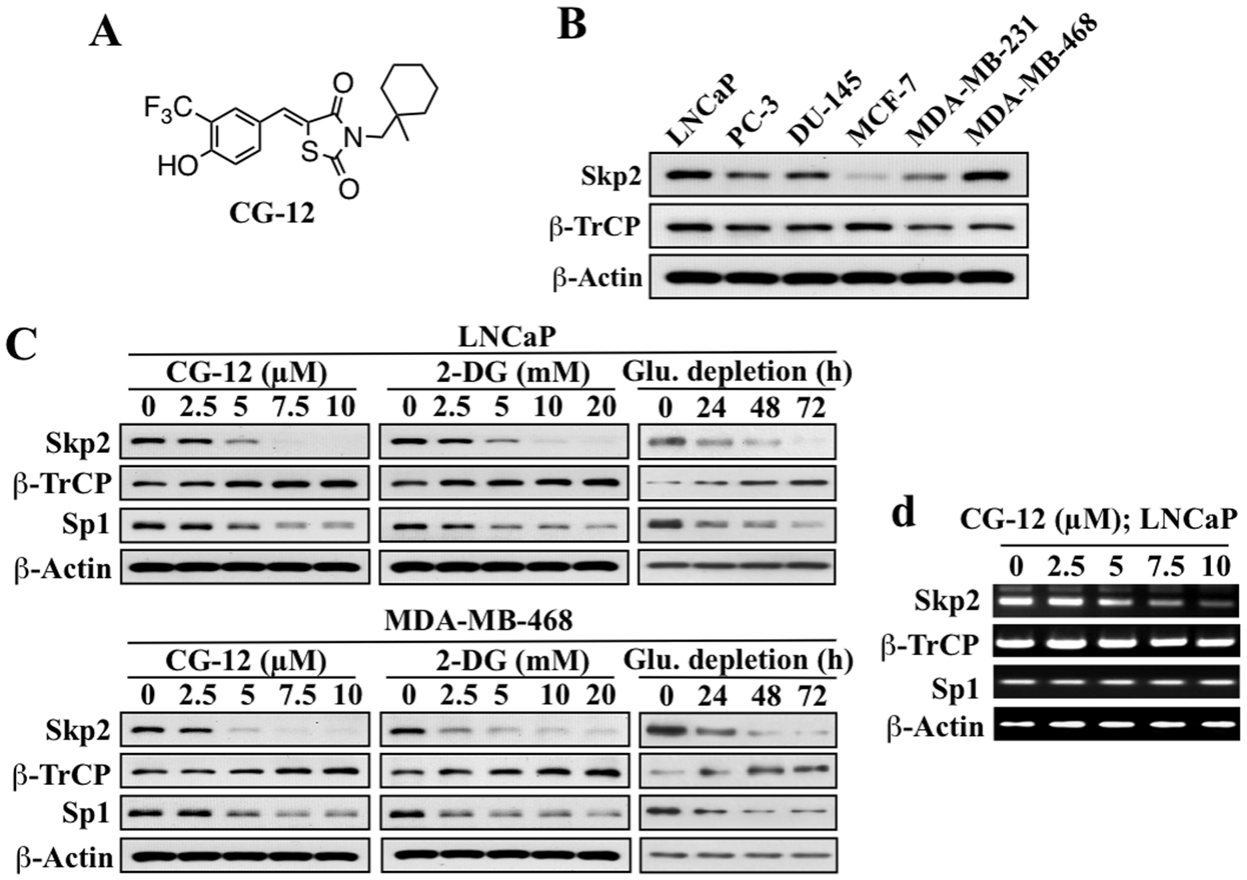
Dichotomous effects of ERMAs and glucose starvation on the expression of Skp2 and β-TrCP. (A) Structure of CG-12. (B) Relative expression levels of Skp2 and β-TrCP in six different prostate and breast cancer cell lines. (C) Western blot analysis of the dose- or time-dependent effects of CG-12, 2-DG, and glucose deprivation on the expression of Skp2, β-TrCP, and Sp1 in LNCaP (upper panel) and MDA-MB-468 cells (lower panel). Cells were exposed to CG-12 or 2-DG at the indicated concentrations in 10% FBS-supplemented medium for 72 h. (D) RT-PCR analysis of the dose-dependent effect of CG-12 on the mRNA expression of Skp2, β-TrCP, and Sp1 in LNCaP cells after 72 h of treatment.

**Figure 2:**
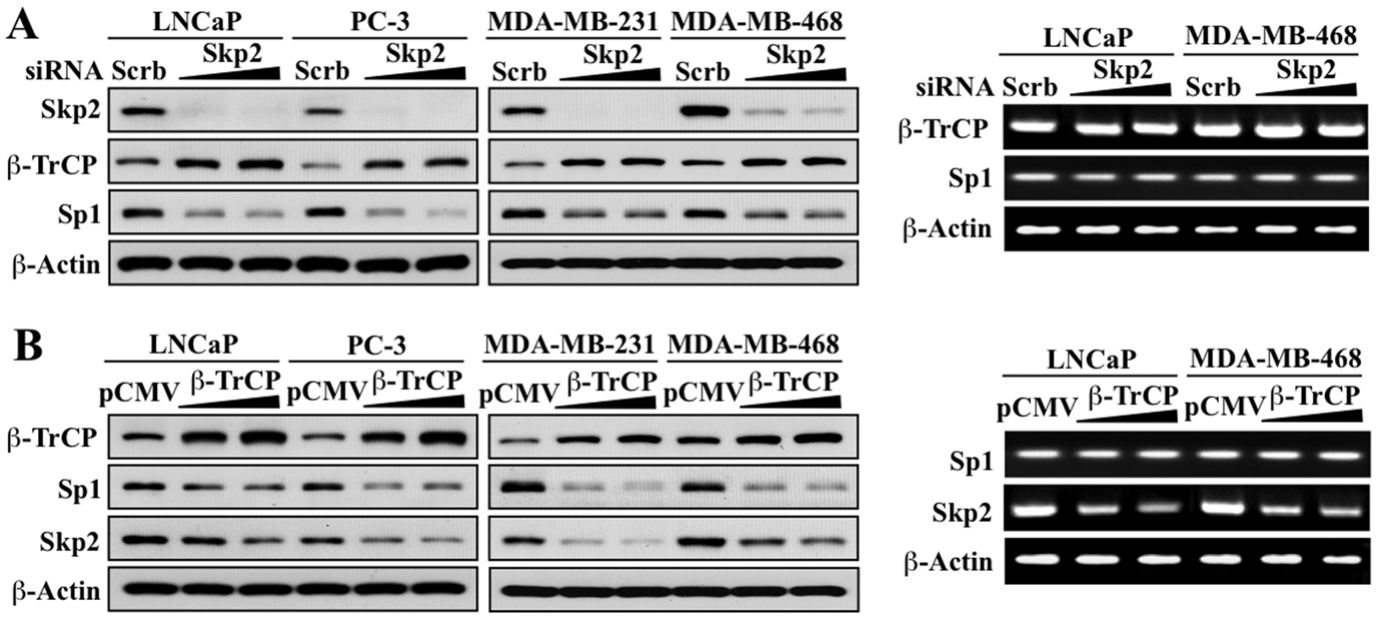
Evidence that Skp2 and β-TrCP are mutually regulated. (A) Western blot (left panel) and RT-PCR (right panel) analyses of the effects of siRNA-mediated knockdown of Skp2 on the expression levels of β-TrCP and Sp1 in LNCaP, PC-3, MDA-MB-231, and/or MDA-MB-468 cells. (B) Western blot (left panel) and RT-PCR (right panel) analyses of the effects of ectopic expression of β-TrCP on the expression of levels of Sp1 and Skp2 in LNCaP, PC-3, MDA-MB-231, and/or MDA-MB-468 cells. Scrb, scrambled siRNA control; pCMV, empty vector control.

Previously, we demonstrated that increased β-TrCP protein expression represents a cellular response to energy restriction induced by ERMAs, including CG-12 (structure, Fig. 1A) and 2-deoxyglucose (2-DG), or glucose depletion in cancer cells, from which the resulting degradation of cell cycle- and proliferation-regulatory proteins plays a crucial role in triggering downstream apoptosis signaling [18]. Thus, this study aimed to define the mechanism by which energy restriction induces this accumulation of β-TrCP in cancer cells. Our results demonstrate that Skp2 is the E3 ligase responsible for β-TrCP degradation, and that energy restriction-induced β-TrCP accumulation resulted from decreased Skp2 expression consequent to Sirt1-induced downregulation of COP9 signalosome (Csn)5 expression. Equally important, we obtained evidence for a novel feedback regulation between Skp2 and β-TrCP, which amplified the effect of energy restriction on Skp2 downregulation. Specifically, as reduced Skp2 expression gave rise to β-TrCP accumulation, the consequent decrease in protein levels of Sp1, a β-TrCP substrate, resulted in decreased Skp2 gene expression. This feedback loop represents a novel crosstalk mechanism between these two important F-box proteins in cancer cells with aberrant Skp2 expression under energy restriction, which provides a proof-of-concept that the oncogenic Csn5/Skp2 signaling axis represents a “druggable” target.

## Materials and Methods

### Cell Culture and Reagents

Prostate cancer cells, LNCaP, PC3 and DU-145, and breast cancer cells, MCF-7, MDA-MB-231 and MDA-MB-468, were obtained from the American Type Culture Collection (Manassas, VA). RPMI1640 and DMEM/F12 medium supplemented with 10% fetal bovine serum (FBS) were used maintain prostate and breast cancer cells, respectively. CG-12 was synthesized according to a published procedure [19], and 2-DG, nicotinamide, splitomicin, and roscovitine were purchased from Sigma-Aldrich (St. Louis, MO). The kinase inhibitors SB216763, PD98059, and SP600125 were purchased from LC Laboratories (Woburn, MA). Antibodies specific for the following proteins were used in the study. Mouse monoclonal antibodies: β-TrCP, Skp2, and Fbw7, Invitrogen (Carlsbad, CA); Myc, Cell Signaling Technology (Beverly, MA); Cdh1, Millipore (Billerica, MA); Fbx2, Fbxw8, Novus Biologicals (Littleton, CO); β-actin, MP Biomedicals (Irvine, CA). Rabbit antibodies: Emi1, Millipore; Fbx4, Rockland (Gilbertsville, PA); Fbx7, Fbx31, Novus Biologicals; Flag, Nedd8 and Sirt1, Cell Signaling; hemagglutinin (HA), Sp1 and Csn5, Santa Cruz Biotechnology. The Flag-tagged wild-type and H363Y dominant–negative Sirt1, and HA-tagged Sirt1 plasmids were purchased from Addgene (Cambridge, MA). Flag-tagged Cul1 and Myc-tagged β-TrCP, Skp2, and Fbw7 plasmids were previously described [12]. Sp1 siRNA was purchased from Upstate (Lake Placid, NY).

### Plasmid Construction and Site-directed Mutagenesis

The cDNA encoding full-length human Csn5 was PCR-amplified from a human testicular cDNA library with primers, 5′-CGGAATTCTATGGCGGCGTCCGGGAGC-3′ (forward) and 5′-CGGGATCCTTA-AGAGATGTTAATTTG-3′ (reverse), that were flanked by EcoRI and BamHI restriction sites. The resulting fragments were subcloned into the p3XFLAG-CMV26 expression vector. Plasmids encoding various mutations on Csn5, Cul1, and β-TrCP were generated by site-directed mutagenesis using the QuickChange site-directed mutagenesis kit from Stratagene (Cedar Creek, TX). Primers used were as follows:

D151N Csn5∶5′-TCTGGGATTAATGTTAGTACTCAG -3′ and 5′-CTGAGTACTAA-CATTAATCCCAGA -3′; K720A Cul1∶5′-GTGAGAATCATGGCGATGAGGAAGG-TT-3′ and 5′-AACCTTCCTCATCGCCATGATTCTCAC-3′; S412A β-TrCP: 5′-TGGGATATGGCCGCCCCAACTGACATT-3′ and 5′-AATGTCAGTTGGGGCGGC-CATATCCCA-3′; S595A β-TrCP: 5′-GAACCCCCCCGTGCCCCTTCTCGAACA-3′ and 5′-TGTTCGAGAAGGGGCACGGGGGGGTTC-3′.

Truncated constructs of β-TrCP and Skp2 were generated from the β-TrCP- and Skp2-Myc plasmids using the following primers:

ΔCT-β-TrCP: 5′-CCGATATAAGCTTATGGACCCGGCCGAG-3′ and 5′-GCTCTAGACTTCTTCCACAGCAT-3′; ΔF-Skp2∶5′-ATGAATTCATTAGACCTCA-CAGGTAA-3′ and 5′- AAGATATCCTTAGACAACTGGGCTTTTGC-3′; ΔLRR-Skp2∶5′-ATGAATTCAATGCACAGGAAGCACCTCCAGG-3′ and 5′-AAGATATCCTTAA-GGTCTGCCATAGAG-3′; ΔNT-Skp2∶5′-ATGAATTCATCCCTTCCGGATGAGCT-3′ and 5′-AAGATATCCTTAGACAACTGGGCTTTTGC-3′. ΔF-β-TrCP and ΔNT-β-TrCP were generated as previously described [13]. The resulting fragments were ligated into the pMyc4-CMV14 expression vector. The Skp2 promoter-luciferase plasmids including WT and mutated Sp1 A and B binding sites and YY1 and GATA-1 sites were described previously [20]. All constructs were verified by DNA sequencing.

### RNA Isolation and Semi-quantitative PCR Analysis

Total RNA was isolated from drug-treated LNCaP cells using an RNeasy mini kit (Qiagen, Valencia, CA), and reverse-transcribed to cDNA using Omniscript RT Kit (Qiagen) according to manufacturer’s instruction. PCR primers used were as follows: Csn5, 5′-ATATCCGCAGGGAAAG-3′ and 5′-GGTCCTTCATCAGGAGGTTTGT-3′; Sirt1, 5′-GAACAGGTTGCGGGAATC-3′ and 5′-AACATGAAGAGGTGTGGGTG-3′; Sp1, 5′-GGCGAGAGGCCATTTATG-TGT-3′ and 5′-AGTGGCATCAACGTCATGCA-3′; β-TrCP, 5′-CACTTAGACACAT-ACAACA-3′ and 5′-TCTGCAACATAGGTTTAAGAT-3′; Skp2, 5′-CCTAAGCAGCT-GTTCCC-3′ and 5′-TTCGAGATACCCACAACCCC-3′; β-actin, 5′-TCTACAATGAG-CTGCGTGTG-3′ and 5′-GGTCAGGATCTTCATGAGGT-3′. PCR products were separated electrophoretically in 1% agarose gels and visualized by ethidium bromide staining.

### Transient Transfection, Immunoprecipitation and Immunoblotting

Transfections were performed by electroporation using the Amaxa Nucleofector with Nucleofector kit R (Amaxa Biosystems, Cologne, Germany). Cell lysates were harvested with M-PER lysis buffer (Pierce, Rockford, IL). Immunoprecipitation and immunoblotting for various target proteins were performed as previously described [18].

### Chromatin Immunoprecipitation (ChIP) Assay

CG-12 (5 µM)-treated LNCaP cells were fixed with 1% formaldehyde at 37°C for 10 min. Cells were washed twice with ice-cold PBS containing protease inhibitors (1 mM phenylmethylsulphonyl fluoride, 1 mg/ml aprotinin, and 1 mg/ml pepstatin A), harvested by scraping and pelleted by centrifugation at 4°C. Cells were resuspended in a lysis buffer (1% SDS, 10 mM EDTA, and 50 mM Tris-HCl, pH 8.1), incubated for 10 min on ice, and sonicated to shear DNA. After sonication, lysate was centrifuged for 10 min at 13,000 rpm at 4°C. The supernatant was diluted in ChIP dilution buffer (0.01% SDS, 1% Triton X-100, 2 mM EDTA, 16.7 mM Tris-HCl, pH 8.1, 167 mM NaCl, and protease inhibitors). Anti-Sp1 antibody was added to the supernatant and incubated overnight at 4°C with rotation. DNA fragments were recovered and were subjected to PCR amplification using primers specific for the detection of the −421/-222 region of the human *SKP2* promoter which contains the Sp1 binding sites. The sequences of the primers used are: 5′-AGAAAGGAAAATCCGTCTAC-3′ and 5′-TAGTTTCCGTCCCGCTTCGT-3′. The size of the PCR product was 200 bp.

### Promoter Activity Assay

LNCaP cells expressing wild type and mutant *SKP2* promoter-luciferase and thymidine kinase promoter-*Renilla reniformis* luciferase constructs were treated with DMSO or 5 µM CG-12 for 48 h. Reporter gene assays were then performed as we reported previously [13].

## Results

### Interplay between Skp2 and β-TrCP in Response to Energy Restriction

Analysis of the expression levels of Skp2 and β-TrCP in different prostate and breast cancer cell lines, including LNCaP, PC-3, DU-145, MCF-7, MDA-MB-231, and MDA-MB-468, revealed that these F-box proteins are differentially expressed among these cells (Fig. 1B). Consistent with Skp2’s role as a downstream effector of androgen receptor in mediating proliferation in prostate cancer cells [21], its expression level in androgen-responsive LNCaP cells is substantially higher than in androgen-independent PC-3 and DU-145 cells. In breast cancer cells, Skp2 expression is associated with lack of estrogen receptor (ER) and HER-2 [22], which is reflected by the elevated expression levels of Skp2 in the triple-negative breast cancer cell lines MDA-MB-468, and, to a lesser extent, MDA-MB-231, relative to ER-positive MCF-7 cells. Compared to Skp2, β-TrCP is ubiquitously expressed across these cell lines.

Previously, we reported that exposure of LNCaP cells to ERMAs, including the aforementioned CG-12, as well as to glucose depletion, led to increases in β-TrCP expression through protein stabilization, of which the underlying mechanism remained undefined [12], [13]. It is noteworthy that this drug-induced β-TrCP upregulation was correlated with reduced Skp2 expression, while other F-box proteins examined, including Fbw7, Fbx4, and Fbxw8, were less affected [12], [13]. This dichotomous effect on the expression of β-TrCP and Skp2 suggested a possible link between these two F-box proteins in response to energy restriction. To shed light onto this issue, we examined the effects of the ERMAs, 2-DG and CG-12, and glucose deprivation on the expression of Skp2, β-TrCP, and Sp1, a β-TrCP target protein, in LNCaP and MDA-MB-468 cells, both of which express abundant levels of Skp2. Reminiscent of our previous reports [12], [13], these treatments led to dose- and time-dependent reductions in Skp2 expression, accompanied by parallel increases and decreases in the levels of β-TrCP and Sp1, respectively (Fig. 1C). RT-PCR analysis in CG-12-treated LNCaP cells indicates that this suppression of Skp2 expression also occurred at the mRNA level, although to a lesser extent than that of the protein levels (Fig. 1D), suggesting the involvement of both transcriptional and posttranslational mechanisms. In contrast, CG-12-induced changes in β-TrCP and Sp1 protein levels were not associated with corresponding alterations in mRNA expression.

Next, we obtained evidence that the opposing changes in the expression of Skp2 and β-TrCP in response to energy restriction represent a causal relationship by evaluating the effect of siRNA-mediated knockdown of Skp2 or ectopic expression of β-TrCP on the expression of the respective counterparts, as well as Sp1, in LNCaP, PC-3, MDA-MB-231, and MDA-MB-468 cells. Western blot analysis indicates that siRNA-mediated knockdown of Skp2 in these cells led to increased expression of β-TrCP, and parallel decreases in Sp1 expression (Fig. 2A, left panel). These changes, however, were not noted at the mRNA level in LNCaP and MDA-MB-468 cells (Fig. 2A, right panel), suggesting a link between Skp2 and β-TrCP protein stability. The enforced expression of β-TrCP in the same cell lines decreased the protein expression of its substrate Sp1 as expected, while causing parallel decreases in Skp2 expression at both the protein and mRNA levels (Fig. 2B), suggesting that ectopic β-TrCP expression resulted in the transcriptional repression of Skp2.

Together, these findings indicate an intricate relationship between Skp2 and β-TrCP in energy-restricted cancer cells in which downregulated Skp2 expression is involved in the glycolysis inhibitor-induced accumulation of β-TrCP, which in turn contributes to the reduced expression of Skp2. To more thoroughly understand this interplay, we investigated the mechanism by which inhibited glycolysis suppressed Skp2 expression.

### Sirt1 Plays a Key Role in Mediating the Effect of ERMAs on Skp2 and β-TrCP Expression by Down-regulating Csn5

**Figure 3:**
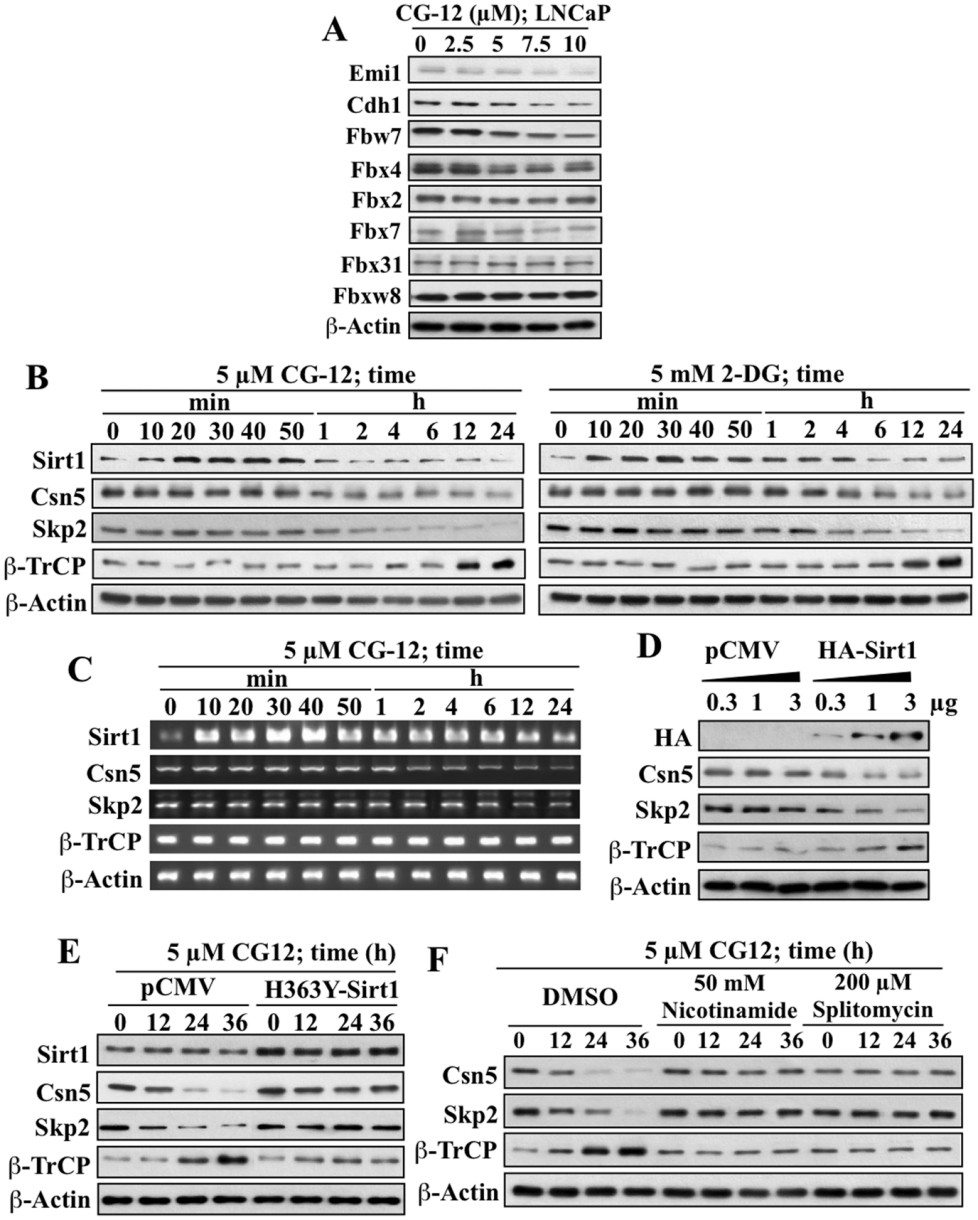
Evidence that Sirt1 mediates the effects of energy restriction on Skp2 and β-TrCP expression through Csn5 downregulation. (A) Western blot analysis of the dose-dependent effects of CG-12 on the expression of Emi1, Cdh1, and various F-box proteins related to cell proliferation, including Fbw7, Fbx2, Fbx4, Fbx7, Fbx31, and Fbxw8, after 72 h of treatment. (B) Western blot analysis of the time-dependent effects of 5 µM CG-12 (left panel) and 5 mM 2-DG (right panel) on the expression levels of Sirt1, Csn5, Skp2, and β-TrCP. (C) RT-PCR analysis of time-dependent effects of 5 µM CG-12 on the mRNA expression levels of Sirt1, Csn5, Skp2, and β-TrCP. (D) Ectopic expression of Sirt1 mimicked the effects of ERMAs on the expression of Csn5, Skp2 and β-TrCP proteins. (E) Dominant-negative inhibition of Sirt1 by ectopically expressed H363Y-Sirt1 protected cells from the effect of CG-12 on the expression of Csn5, Skp2 and β-TrCP proteins. (F) Pharmacological inhibition of Sirt1 deacetylase activity by nicotinamide and splitomycin protected cells from the effect of CG-12 on the expression of Csn5, Skp2 and β-TrCP proteins.

As aforementioned, Skp2 protein levels can be downregulated via APC/C^Cdh1^-mediated degradation [16], [17]. Nonetheless, two lines of evidence argued against the involvement of APC/C^Cdh1^ in ERMA-induced Skp2 degradation. First, CG-12 at 5 µM substantially reduced Skp2 levels, but virtually had no effect on the expression of Emi1, an endogenous regulator of APC/C^Cdh1^ (Fig. 3A). Second, CG-12 caused a modest downregulation of Cdh1 expression. Moreover, the effects of CG-12 on the expression of Skp2 and β-TrCP were specific, as many other F-box proteins involved in cell proliferation, including Fbw7, Fbx2, Fbx4, Fbx7, Fbx31, and Fbxw8, showed either modest decreases or no changes in their expression levels (Fig. 3A).

Sirt1 gene expression represents a hallmark response to energy restriction [23], and our previous study showed that the transient induction of Sirt1 in response to CG-12 and 2-DG protected β-TrCP from ubiquitin-dependent degradation via a yet-to-be-identified E3 ligase [18]. Moreover, recent reports demonstrate that Csn5, a subunit of the COP9 signalosome, regulates the stability of Skp2 protein through the deneddylation of the Cul1 subunit of SCF [24], which promotes the assembly of a functional SCF^Skp2^ complex by preventing the auto-ubiquitination of Skp2 [25]. Consequently, silencing of Csn5 expression results in increased Cul1 neddylation accompanied by Skp2 degradation [24]. Based on these findings, we hypothesized that Csn5 played an intermediary role between Sirt1 induction and changes in the expression levels of Skp2 and β-TrCP in response to energy restriction.

This premise was corroborated by the temporal relationship between the changes in expression levels of Sirt1 and those of Csn5, Skp2, and β-TrCP in response to 5 µM CG-12 or 5 mM 2-DG in LNCaP cells (Fig. 3B). As shown, the early drug-induced transient increase of Sirt1 preceded the reduction in Csn5 expression, which was accompanied by parallel changes in Skp2 and β-TrCP levels. RT-PCR analysis in CG-12-treated cells indicates that, with the exception of β-TrCP, changes in mRNA expression of the other three signaling molecules were also noted (Fig. 3C). As aforementioned, the magnitude of the reduction in Skp2 mRNA levels was smaller than that of its protein counterpart.

To confirm that Sirt1 induction underlay the ability of CG-12 to modulate the expression of Csn5, Skp2, and β-TrCP, we examined the effect of manipulating Sirt1 status on the expression of these signaling effectors in CG-12-treated LNCaP cells. Ectopic expression of Sirt1 mimicked CG-12’s ability to decrease Csn5 expression, which was paralleled by changes in the levels of Skp2 and β-TrCP (Fig. 3D). In contrast, dominant-negative inhibition of Sirt1 by the deacetylase-defective mutant H363Y-Sirt1 [26] blocked drug-mediated effects on the expression of these signaling effectors (Fig. 3E). The involvement of the deacetylase activity of Sirt1 was confirmed by the ability of its pharmacological inhibitors, nicotinamide (50 mM) and splitomicin (200 µM), to rescue Csn5, Skp2 and β-TrCP from CG-12-induced changes to their expression levels (Fig. 3F).

**Figure 4:**
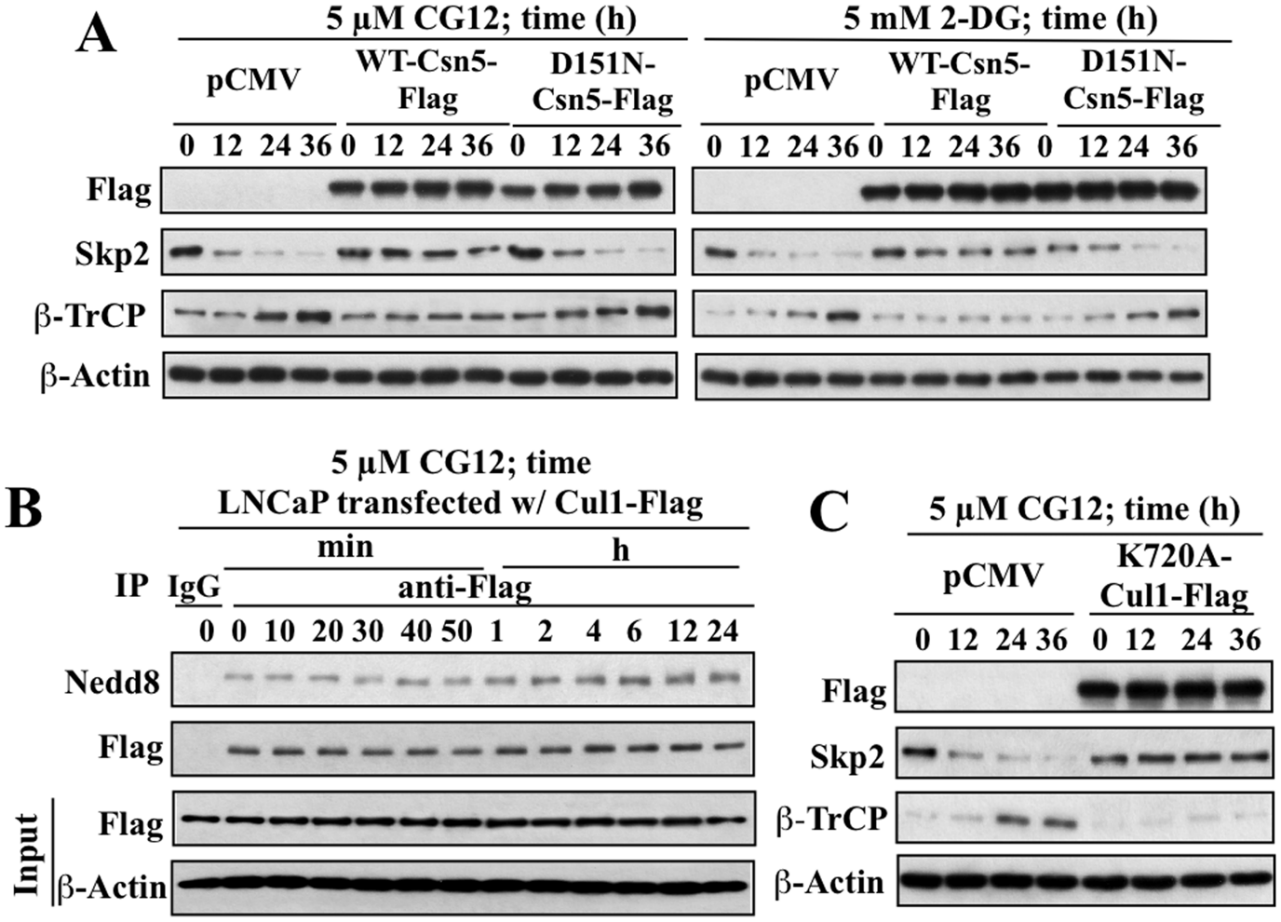
Evidence that Csn5 downregulation mediates the effects of energy restriction on Skp2 and β-TrCP expression through increased Cul1 neddylation. (A) Overexpression of wild-type Csn5, but not the deneddylase-deficient D151N mutant, protected against the time-dependent effects of CG-12 (left panel) and 2-DG (right panel) on the expression of Skp2 and β-TrCP proteins. (B) Exposure to 5 µM CG-12 gave rise to a time-dependent increase in Cul1 neddylation, which paralleled changes in the expression levels of Skp2 and β-TrCP shown in Fig. 3B. (C) CG-12-mediated changes in Skp2 and β-TrCP expression were blocked by ectopically expressed K720A-Cul1, a dominant-negative form of Cul1 lacking the Nedd8 modification site.

The above finding suggests the intermediary role for Csn5 in mediating Sirt1-induced Skp2 repression in response to ERMAs, which is corroborated by several lines of evidence in LNCaP cells (Fig. 4). First, ectopic expression of wild-type Csn5, but not that of the D151N mutant with inactivated deneddylase/isopeptidase activity [27], protected against the time-dependent effects of CG-12 and 2-DG on the expression of Skp2 and β-TrCP (Fig. 4A). Second, Csn5 has been reported to regulate Skp2 stability through the deneddylation of the Cul1 subunit of SCF. As shown in Fig. 4B, CG-12 caused a time-dependent increase in the neddylation level of Cul1, which paralleled the reduction of Skp2 expression. Third, the role of Nedd8 modification of Cul1 in mediating the effects of CG-12 was confirmed by the use of ^K720A^Cul1, a mutant Cul1 lacking the Nedd8 modification site [28], which abolished the ability of CG-12 to alter the expression levels of Skp2 and β-TrCP (Fig. 4C).

### β-TrCP is Targeted by Skp2 for Degradation

**Figure 5:**
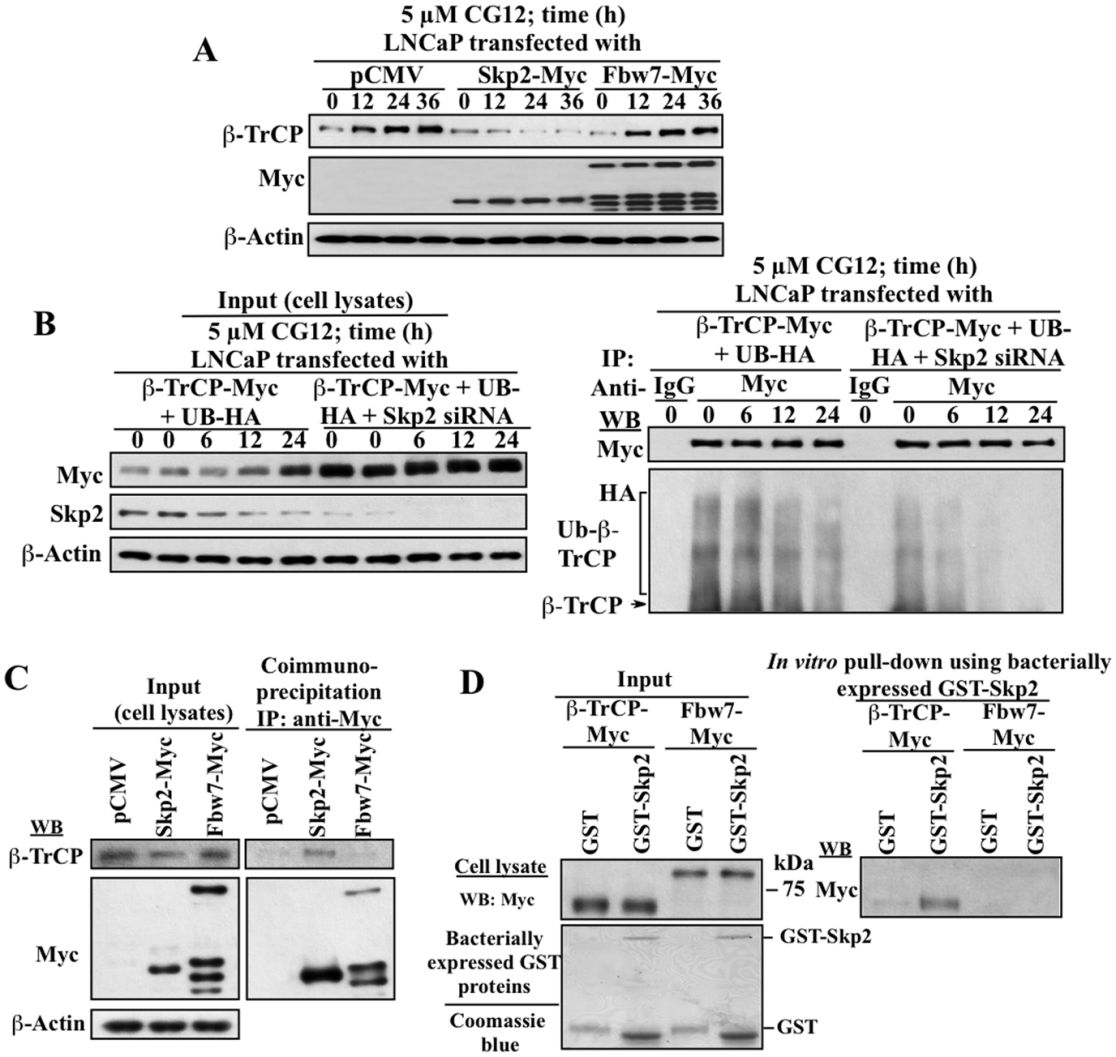
Evidence that β-TrCP is a substrate of Skp2. (A) Ectopic expression of Skp2 abrogated CG-12-induced upregulation of β-TrCP protein expression, while the ectopic expression of the negative control Fbw7 showed no appreciable effect. (B) siRNA-mediated silencing of Skp2 enhanced the suppressive effect of CG-12 on β-TrCP ubiquitination. LNCaP cells were co-transfected with plasmids encoding β-TrCP-Myc and ubiquitin (UB)-HA, with or without Skp2 siRNA, and then treated with 5 µM CG-12 in 10% FBS-supplemented medium for the indicated time intervals. Equal amounts of cell lysates were probed with anti-Myc or anti-Skp2 antibodies (left panel; input) or immunoprecipitated (IP) with anti-Myc affinity gels, followed by immunoblotting (WB) with anti-Myc or anti-HA antibodies (right panel). (C) Pull-down analysis of the selective binding of β-TrCP to Skp2 versus Fbw7. LNCaP cells were nucleofected with plasmids encoding Skp2-Myc or Fbw7-Myc, or with empty pCMV vector. Equal amounts of cell lysates were probed with anti-β-TrCP or anti-Myc antibodies (left panel; input) or immunoprecipitated (IP) with anti-Myc affinity gels, followed by immunoblotting (WB) with anti-β-TrCP or anti-Myc antibodies (right panel). (D) *In vitro* pull-down of β-TrCP-Myc versus Fbw7-Myc by bacterially expressed GST-Skp2. LNCaP cells were transfected with plasmids encoding β-TrCP-Myc or Fbw7-Myc, and equal amounts of cell lysates were incubated with bacterially expressed GST, or GST-Skp2 immobilized onto glutathione beads. The resulting complexes were washed, centrifuged and subjected to Western blot analysis (WB) with anti-Myc antibodies (right panel). One tenth volume of each cell lysate was collected as input and then probed with anti-Myc antibodies, and used to confirm purity and integrity of the recombinant GST-fusion proteins (GST and GST-Skp2) with GST antibody (left panel).

We further established the causal relationship between Skp2 downregulation and CG-12-mediated β-TrCP accumulation by demonstrating that Skp2 targeted β-TrCP for degradation. As shown, ectopic expression of Skp2 abrogated the effect of CG-12 on β-TrCP expression in LNCaP cells, indicating a role for Skp2 downregulation in CG-12-mediated β-TrCP accumulation. This protective effect, however, was not observed with the enforced expression of the negative control Fbw7 (Fig. 5A). To examine the effect of CG-12 on β-TrCP ubiquitination and the role of Skp2 in this process, LNCaP cells were co-transfected with Myc-tagged β-TrCP (β-TrCP-Myc) and HA-tagged ubiquitin (UB-HA) in the presence or absence of Skp2 siRNA and the level of β-TrCP ubiquitination was determined by co-immunoprecipitation (Fig. 5B). As shown, exposure of these cells to 5 µM CG-12 led to a time-dependent decrease in ubiquitinated β-TrCP levels. It is noteworthy that siRNA-mediated silencing of Skp2 shared the ability of CG-12 to suppress β-TrCP ubiquitination (time = 0), and enhanced the time-dependent suppressive effect of CG-12 on β-TrCP ubiquitination. Furthermore, the physical interaction between Skp2 and β-TrCP was verified in two ways: co-immunoprecipitation of β-TrCP with ectopically expressed Myc-tagged Skp2 (Skp2-Myc) versus Fbw7-Myc in LNCaP cells (Fig. 5C), and *in vitro* pull-down of β-TrCP-Myc versus Fbw7-Myc in LNCaP cell lysates by bacterially expressed GST-Skp2 (Fig. 5D). As shown, both analyses demonstrated the binding of β-TrCP with Skp2, which, however, was not noted with Fbw7.

### Identification of the Skp2-recognition Motif in β-TrCP

**Figure 6:**
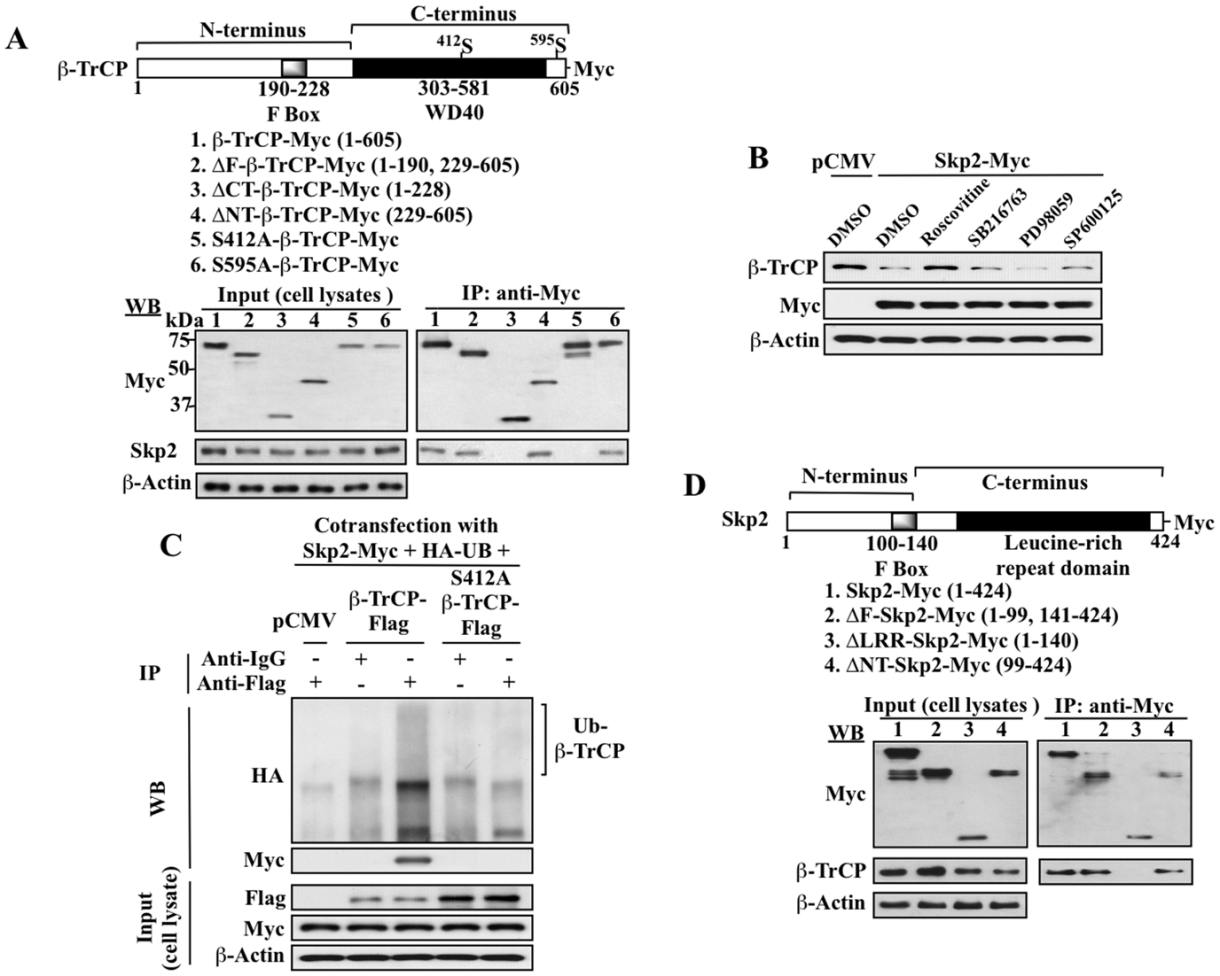
Identification of the Skp2 recognition motif in β-TrCP and the β-TrCP-interacting region in Skp2. (A) Upper panel, schematic representation of the structures of the wild type and mutant forms of β-TrCP-Myc. Lower panels, pull-down analysis of the binding of different mutant forms of β-TrCP-Myc versus the wild-type β-TrCP to endogenous Skp2 in LNCaP cells. LNCaP cells were nucleofected with plasmids encoding wild type (1) or mutated forms (2–6) of β-TrCP-Myc. Equal amounts of cell lysates were probed with anti-Myc or anti-Skp2 antibodies (left panel; input) or immunoprecipitated (IP) with anti-Myc affinity gels, followed by immunoblotting (WB) with anti-Myc or anti-Skp2 antibodies (right panel). (B) Identification of the kinase responsible for the proline-directed phosphorylation of Ser-412 required for Skp2-mediated β-TrCP degradation. LNCaP cells ectopically expressing Skp2-Myc were exposed to DMSO vehicle, the CDK2 inhibitor roscovitine (10 µM), the GSK3β inhibitor SB216763 (10 µM), the ERK inhibitor PD98059 (25 µM), and the JNK inhibitor SP600125 (10 µM) for 24 h. Cell lysates were immunoblotted with anti-β-TrCP and anti-Myc antibodies. (C) The S412A mutation of β-TrCP blocks Skp2 recognition and Skp2-mediated ubiquitination of β-TrCP. LNCaP cells were co-transfected with plasmids encoding Skp2-Myc, ubiquitin (UB)-HA, and the wild type or S412A form of β-TrCP-Flag versus pCMV, and incubated for 24 h. Equal amounts of cell lysates were probed with anti-Myc or anti-Flag antibodies (lower panel; input) or immunoprecipitated (IP) with anti-Flag affinity gels, followed by immunoblotting (WB) with anti-HA or anti-Myc antibodies (upper panel). (D) Identification of the β-TrCP-interacting region of Skp2 by mutational analysis of Skp2 binding to β-TrCP. Upper panel, schematic representation of the structures of wild type and truncated forms of the Skp2-Myc fusion protein. Lower panels, pull-down analysis of the binding of wild type versus different truncated forms of Skp2-Myc to endogenous β-TrCP in LNCaP cells. Wild type (1) and truncated forms (2–4) of Skp2-Myc were ectopically expressed in LNCaP cells. Equal amounts of cell lysates were probed with anti-Myc or anti-β-TrCP antibodies (left panel; input) or immunoprecipitated (IP) with anti-Myc affinity gels, followed by immunoblotting (WB) with anti-Myc or anti-β-TrCP antibodies (right panel).

To verify that the direct interaction between Skp2 and β-TrCP occurs through specific recognition, mutational analyses were performed to identify the Skp2-recognition motif in β-TrCP. The initial analysis was conducted by examining the binding of a series of truncated mutants of β-TrCP1-Myc, including those with a deleted F-box (ΔF-β-TrCP1-Myc), a deleted C-terminus (ΔCT-β-TrCP1-Myc), and a deleted N-terminus (ΔNT-β-TrCP1-Myc), vis-à-vis the wild-type counterpart to endogenous Skp2 in LNCaP cells by the Myc pull-down assay (Fig. 6A). While deletion of the F-box or N-terminus had no effect on the binding of β-TrCP to Skp2, removal of the C-terminus abrogated its interaction with Skp2, thereby indicating the presence of a Skp2 recognition site within this domain.

Skp2 substrates lack an apparent consensus sequence for the Skp2 “degron”, thus, to more thoroughly characterize the β-TrCP motif responsible for Skp2 recognition, we analyzed the sequences surrounding the reported phosphorylation site(s) in a series of Skp2 substrates, including p27, p57, p130, RAG, TAL1, MKP1, E2A, and MEF, and identified the relevant kinases [29], [30], [31], [32], [33], [34], [35], [36] (Table 1). Comparison of these sequences reveals a consensus SP/TP motif for phosphorylation-dependent Skp2 recognition. This specific phosphorylation of Ser or Thr that immediately precedes a proline residue is characteristic of a group of proline-directed kinases, including cyclin-dependent protein kinases (CDKs), mitogen-activated protein kinases, N-terminal protein kinases (JNKs), and glycogen synthase kinase-3 (GSK-3) [37]. For the various Skp2 substrates listed in Table 1, the phosphorylation of this SP/TP motif was mediated through either CDK2 or extracellular signal-regulated kinase (ERK). Thus, we investigated the role of proline-directed kinases in regulating Skp2-mediated β-TrCP degradation by exposing LNCaP cells transiently transfected with plasmids encoding Skp2-Myc to inhibitors of CDK2 (roscovitine, 10 µM), GSK3β (SB216763, 10 µM), ERK (PD98059, 25 µM), and JNK (SP600125, 10 µM). As shown in Fig. 6B, relative to the pCMV control, ectopic expression of Skp2 led to the expected downregulation of β-TrCP, which, however, could be rescued by roscovitine, but not the other three inhibitors examined, indicating a link between CDK2 and Skp2-mediated β-TrCP degradation.

**Table 1:** Comparison of the sequences surrounding the reported phosphorylation site(s) in various Skp2 substrates and the identities of kinases involved in their Skp2-mediated degradation.

Substrate	Sequences surrounding the phosphorylation site(s)	Responsible kinase
p27	GSVED^187^ **TP**KKPG	N/A^a^
p57	GSVEQ^310^ **TP**RKRL	CDK2
p130	YDRYS^672^ **SP**PAST	CDK2
RAG	ARALH^490^ **TP**QRVL	CDK2
TAL1	LDGAA^300^ **SP**DSYT	N/A^b^
MKP1	RRSII^296^ **SP**NFSFMG; SAEAG^323^ **SP**AMA	ERK
E2A	NNFSS^352^ **SP**S^355^ **TP**VG^359^ **SP**QGLA	ERK
MEF	LTRSP^643^ **TP**APF^359^ **SP**F	CDK2

a Not reported.

b Pharmacological inhibition of CDK, p38, or ERK had no protective effect.

Furthermore, sequence analysis of the C-terminus of β-TrCP1 indicated the presence of two SP/TP motifs: VWDMA^412^SPTDIT and AEPPR^595^SPSRTY. To discern the role of these two putative recognition motifs in Skp2 binding, β-TrCP1-Myc mutants were created by replacing the Ser412 or Ser595 residue with Ala via site-directed mutagenesis. Pull-down analysis of these two mutants for binding to endogenous Skp2 in transfected LNCaP cells indicates that the substitution at Ser412 (S412A), but not that at Ser595 (S595A), abolished the binding of β-TrCP to Skp2 (Fig. 6A, lanes 5 and 6). To confirm the role of Ser412 in Skp2-facilitated β-TrCP ubiquitination and degradation, cells were co-transfected with plasmids encoding Skp2-Myc, HA-UB, and the wild-type or S412A form of Flag-tagged β-TrCP, and the levels of β-TrCP ubiquitination were determined by co-immunoprecipitation. As shown, relative to the wild-type counterpart, S412A-β-TrCP was resistant to Skp2-mediated degradation (Fig. 6C, Input). Moreover, anti-Flag pull-down, followed by Western blot analysis with anti-Myc and HA antibodies, indicates that the S412A mutation abolished the ability of β-TrCP to bind Skp2 and to undergo Skp2-mediated ubiquitination (Fig. 6C, WB). Together, these data suggest that phosphorylation of β-TrCP at Ser412 by CDK2 is required for Skp2-facilitated degradation.

Finally, an additional analysis was conducted to examine the binding of a series of truncated mutants of Skp2-Myc, including those with a deleted F-box (ΔF-Skp2-Myc), a deleted leucine-rich repeat domain (LRR) (ΔLRR-Skp2-Myc), and a deleted N-terminus (ΔNT-Skp2-Myc), vis-à-vis the wild-type Skp2-Myc to endogenous β-TrCP in LNCaP cells (Fig. 6D). As shown, only deletion of the LRR domain abrogated the ability of Skp2 to bind β-TrCP. This finding confirms the involvement of the LRR domain of Skp2 in its interaction with β-TrCP, which is consistent with its previous identification as a recognition motif for substrate binding by Skp2 [38].

### Sp1 Downregulation Mediates a Positive Feedback Loop that Amplifies Energy Restriction-induced β-TrCP Accumulation via the Inhibition of *Skp2* Gene Expression

**Figure 7:**
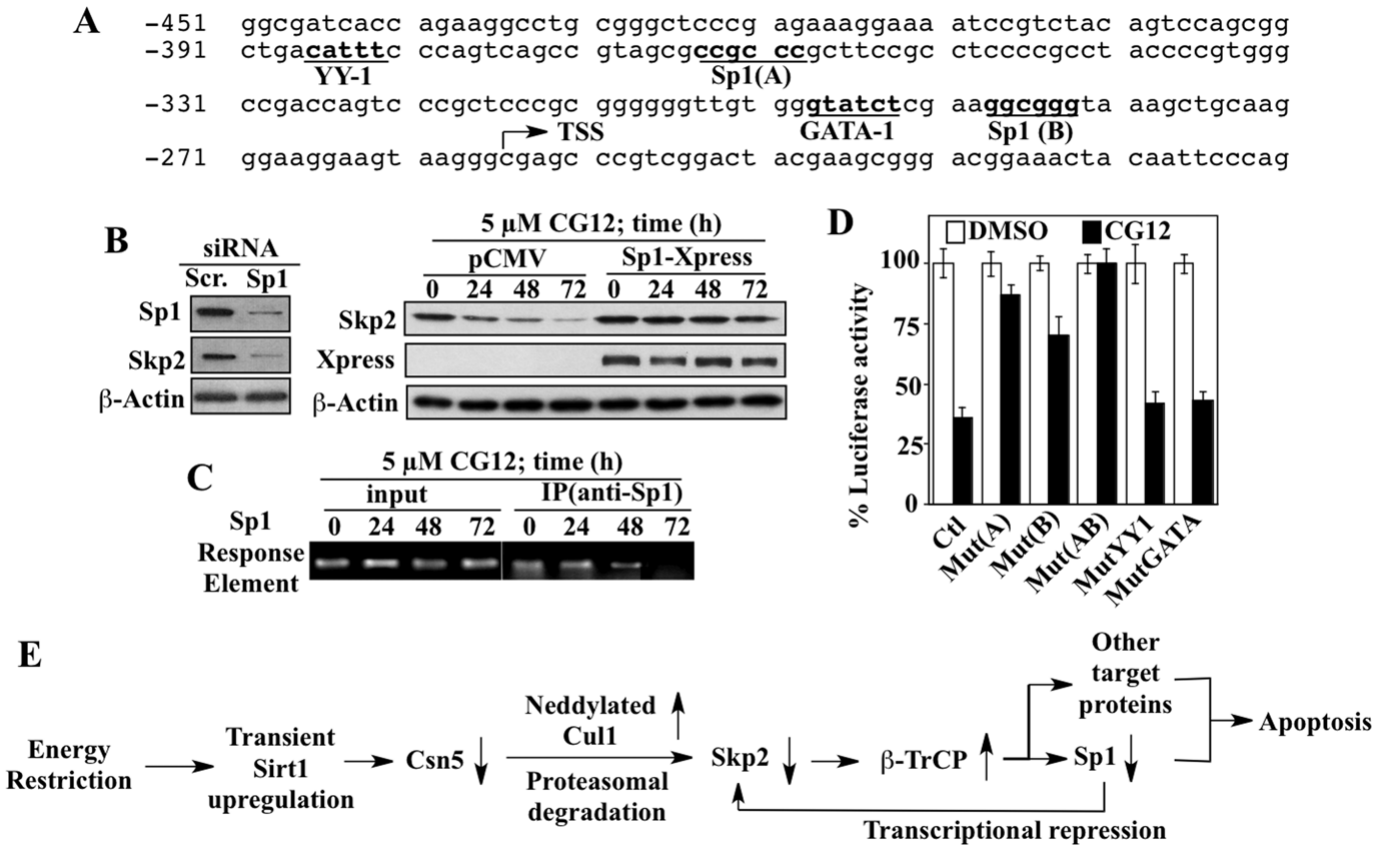
Evidence that Sp1 contributes to the feedback regulation of energy restriction-induced β-TrCP accumulation via Skp2 repression. (A) Sequence of the promoter region of the *SKP2* gene showing two putative Sp1 recognition sites [Sp1(A), Sp1(B)], as well as one putative binding site each for YY-1 and GATA-1. TSS, transcriptional start site. (B) Left panel, siRNA-mediated Sp1 knockdown suppressed Skp2 protein expression in LNCaP cells. Right panel, overexpression of Sp1 protected cells against CG-12-mediated suppression of Skp2 protein expression. (C) Time-dependent decreases in the recruitment of Sp1 to the Skp2 gene promoter in response to CG-12 treatment in LNCaP cells as determined by ChIP analysis. (D) Mutations at the Sp1 A and/or B binding sites, but not at the YY1 or GATA-1 site, abrogated CG-12-mediated suppression of Skp2 promoter transcriptional activity as determined by promoter-luciferase reporter assays. Columns, means (n = 3); bars, S.D. (E) Schematic diagram depicting a novel Skp2-β-TrCP-Sp1 regulatory loop downstream of Sirt1-mediated downregulation of Csn5 in response to energy restriction, culminating in accentuated upregulation of β-TrCP-mediated apoptosis signaling.

Our data indicate that the suppressive effect of ERMAs on Skp2 expression was, in part, attributable to reduced gene expression (Fig. 1D and 3C). As the *SKP2* gene promoter contains two putative Sp1 recognition sites [20] (Fig. 7A), we hypothesized that β-TrCP-mediated Sp1 downregulation might play a role in CG-12-mediated inhibition of Skp2 expression, thereby providing a positive feedback loop for drug-induced β-TrCP accumulation. This premise was corroborated by three lines of evidence. First, siRNA-mediated knockdown of Sp1 led to decreased Skp2 expression in LNCaP cells (Fig. 7B, left panel). Conversely, overexpression of Sp1 increased Skp2 expression (time = 0), and reduced the suppressive effect of CG-12 on Skp2 levels (right panel). Second, chromatin immunoprecipitation (ChIP) analysis indicates that CG-12 treatment decreased the association of Sp1 with the Skp2 promoter DNA in a time-dependent manner (Fig. 7C). Third, abrogation of Sp1 binding to the Skp2 promoter via mutations at the A and/or B Sp1-recognition sites [20] protected against CG-12-mediated inhibition of Skp2 transcription in a promoter-luciferase reporter assay (Fig. 7D). This protective effect, however, was not noted with a mutation at the YY1 or GATA-binding site.

## Discussion

Here, we report a novel Skp2-β-TrCP-Sp1 regulatory loop through which ERMAs up-regulate β-TrCP expression as a major antitumor mechanism of action in cancer cells (Fig. 7E). Induction of Sirt1 represents a hallmark cellular response to glucose starvation, and our data indicate that this induction, though transient and short-lived, led to transcriptional repression of Csn5. This mechanistic link was confirmed by the protective effect of dominant-negative or pharmacological inhibition of Sirt1 on CG-12-induced suppression of Csn5 expression. This Csn5 downregulation is noteworthy considering the oncogenic role of Csn5 in promoting malignant transformation and progression in many types of cancer [39], which is, in part, attributable to upregulating Skp2 expression [24]. In light of the therapeutic potential of targeting Csn5/Skp2 in cancer treatment [40], the ability of CG-12 to downregulate the expression of Csn5 and Skp2 through Sirt1 induction provides a proof-of-concept that the Csn5/Skp2 signaling axis represents a “druggable” target for this novel ERMA.

Equally important, we identified Skp2 as the E3 ligase responsible for β-TrCP degradation, which underlines the complexity in the functional interplay between these two F-box proteins in different cellular contexts. Substantial work has demonstrated the delicate balance between β-TrCP and Skp2 in modulating the turnover of many cell cycle-regulatory proteins during the G1/S transition, in which β-TrCP-facilitated Emi1 degradation promotes the degradation of Skp2 via APC/C^Cdh1^ [16], [17]. As Skp2 is often activated in human cancers to increase cell proliferation through genetic amplification [41] and/or upregulated PI3K/Akt signaling [42], the dynamics of Skp2 and β-TrCP interactions is distinct in cancer cells to maintain protein homeostasis. In this context, this study sheds light onto a distinct regulatory mechanism between these two F-box proteins in Skp2-overexpressing cancer cells in response to glucose inhibitors. As β-TrCP promotes the degradation of many cell cycle- and proliferation-related proteins, the ability of Skp2 to suppress β-TrCP proteolysis bestows a growth advantage on these cancer cells. From a mechanistic perspective, the ability to activate β-TrCP-mediated proteolysis through Skp2 downregulation represents a major pathway by which ERMAs suppress cancer cell proliferation.

The targeted degradation of β-TrCP by Skp2 was verified by several experimental results based on the genetic manipulation of Skp2 expression, co-immunoprecipitation, and *in vitro* pull-down assays (Fig. 5). In contrast to other F-box proteins, Skp2 lacks an apparent consensus sequence for substrate recognition. Nevertheless, analysis of a series of Skp2 substrates suggests a shared proline-directed phosphorylation motif, SP/TP, as the recognition site, of which the phosphorylation is mediated by CDK2 or ERK (Table 1). Based on this analysis, we demonstrated that the ^412^SP motif in β-TrCP plays a pivotal role in Skp2 recognition, and that CDK2 is the responsible kinase.

As Sp1 is a β-TrCP substrate [13], glycolysis inhibitor-induced accumulation of β-TrCP resulted in reduced Sp1 levels, which, in turn, decreased Skp2 expression through reduction in gene expression. From a mechanistic perspective, Sp1 provides a functional link that constitutes a feedback loop to amplify the effect of drug-induced Csn5 downregulation on Skp2 and β-TrCP expression. Although this crosstalk mechanism occurs in a cellular context different from that of cell cycle regulation, it provides a molecular basis to account for the antitumor effects of ERMAs by upregulating β-TrCP-mediated apoptosis signaling in cancer cells with aberrant Skp2 expression.
